# Ki67 and Lymphovascular Invasion as Histopathological Predictors of Residual Cancer Burden After Neoadjuvant Chemotherapy in Breast Cancer: A Retrospective Study

**DOI:** 10.3390/diagnostics16081213

**Published:** 2026-04-18

**Authors:** Bogdan Adrian Carabas, Dana Antonia Țǎpoi, Mariana Costache

**Affiliations:** 1 Doctoral School, Carol Davila University of Medicine and Pharmacy, 020021 Bucharest, Romania; 2 Domina Sana Clinic, 020643 Bucharest, Romania; 3 Department of Pathology, Carol Davila University of Medicine and Pharmacy, 020021 Bucharest, Romania; 4 Department of Pathology, Synevo Romania, 077040 Bucharest, Romania; 5 Department of Pathology, University Emergency Hospital, 050098 Bucharest, Romania

**Keywords:** breast cancer, neoadjuvant chemotherapy, prognostic indicators, Ki67, residual cancer burden

## Abstract

**Background:** Neoadjuvant chemotherapy (NAC) is widely used in the management of stage I–III breast cancer, with tumor regression serving as an important surrogate for long-term outcome. Identifying reliable pathological biomarkers predictive of residual disease remains clinically relevant. **Methods:** We conducted a retrospective cohort study of 165 patients with non-metastatic breast cancer treated with neoadjuvant chemotherapy followed by surgery between 2019 and 2022. Pathological response was assessed using the Residual Cancer Burden (RCB) index. The primary study endpoint was extensive residual disease (RCB-III), defined as the poorest category of tumor regression, indicating treatment resistance. Associations between the Nottingham Score together with other histopathological parameters, immunohistochemical markers (ER, PR, HER2), Ki67 proliferation index, and RCB were analyzed using univariate and multivariable logistic regression. **Results:** In univariate analysis, higher Nottingham scores (OR = 1.807, *p* = 0.0017), negative ER expression (OR = 3.017, *p* = 0.0255), the absence of lymphovascular invasion (OR = 0.1877, *p* = 0.0069) and elevated Ki67 (OR = 1.034, *p* = 0.0003) were significantly associated with RCB III. In multivariable analysis, only Ki67 and lymphovascular invasion remained independent predictors of RCB III, while Nottingham score and ER expression lost statistical significance. Correlation analysis demonstrated strong associations between Nottingham score, Ki67, hormone receptor loss, and tumoral necrosis. **Conclusions:** Ki67 is an independent predictor of poor tumor regression following neoadjuvant chemotherapy and appears to capture much of the prognostic information traditionally attributed to histologic grade and Nottingham score. However, the absence of lymphovascular invasion remains a significant positive prognostic factor. These observations support further investigation into the integration of proliferation markers into multivariable predictive models to improve response stratification in breast cancer.

## 1. Introduction

Breast cancer has a prolonged natural progression, with disease progression and death largely driven by local recurrence and distant metastasis. Breast cancer continues to be a significant contributor to cancer-related mortality, ranking just below lung cancer. Nevertheless, the rates of mortality from breast cancer have experienced a downward trend throughout the past decade. The decrease in death rate has been ascribed to both early screening and improvements in breast cancer therapy, including advancements in administered neoadjuvant chemotherapy (NAC). Over the course of the last decade, novel chemotherapeutic agents have emerged [1,2].

Several studies have consistently demonstrated that adjuvant chemotherapy has advantages for all individuals or subcategories of women diagnosed with treatable breast cancer. Nevertheless, the extent of the benefit is contingent upon the comorbidities, the tumor’s specific traits, and the chosen approach [1,2,3]. Adjuvant chemotherapy could be beneficial for breast cancer patients regardless of their hormone receptor status. Patients with hormone receptor-positive tumors typically have a smaller chance of recurrence after chemotherapy. Nevertheless, chemotherapy in hormone receptor-positive, HER2-negative disease is heterogeneous. In particular, tumors with low proliferative activity (low Ki67) often derive negligible benefit from cytotoxic therapy and may be effectively managed with endocrine therapy alone. Conversely, high Ki67 expression serves as a predictive marker for increased chemosensitivity. Rapidly proliferating tumors, despite their aggressive biological phenotype and poorer long-term prognosis, typically exhibit higher rates of pathological complete response (pCR) compared to slowly proliferating Luminal A-like tumors, which are often resistant to cytotoxic agents [3,4,5].

Neoadjuvant chemotherapy plays a crucial role in the preoperative treatment. In addition to facilitating tumor downstaging and increasing rates of breast-conserving surgery, NAC provides an in vivo assessment of tumor chemosensitivity. Another therapeutic objective of neoadjuvant chemotherapy for stage I–III breast cancer is to completely eradicate micrometastases, thereby reducing the potential for relapse [3,6]. Consequently, identifying pathological and molecular markers that reliably predict tumor response to NAC remains a clinically relevant objective.

The Nottingham Grading System is a modification of the Scarff–Bloom–Richardson grading system. It has been approved by various professional organizations worldwide, including the World Health Organization, American Joint Committee on Cancer, European Union, and the Royal College of Pathologists (UK RCPath). This score evaluates glandular/tubular differentiation, nuclear pleomorphism, and mitotic count [6,7,8,9,10]. However, these parameters mainly reflect tumor morphology rather than underlying tumor biology, and the predictive value in neoadjuvant settings remains complex and multifactorial. In this context, immunohistochemical (IHC) markers, including estrogen receptor (ER), progesterone receptor (PR), HER2/neu and Ki67, are routinely assessed in clinical practice to inform prognosis and therapeutic decisions [11,12,13]. Ki67, a biomarker reflecting cellular proliferation, is strongly associated with tumor aggressiveness and clinical outcome [4]. Nevertheless, Ki67 expression is closely intertwined with histologic grade and hormone receptor status, raising questions regarding its independent predictive value. Furthermore, the interactions between these biomarkers, their correlation with the Nottingham Score, and their impact on residual cancer burden remain less well understood.

Tumor regression following neoadjuvant therapy can be objectively quantified using the Residual Cancer Burden (RCB) classification system, which integrates residual tumor size, cellularity, and lymph node involvement. RCB has demonstrated strong prognostic significance and provides a standardized framework for assessing treatment response [14,15,16,17,18,19,20,21,22]. Neoadjuvant chemotherapy induces profound morphological alterations in breast cancer tissue, ranging from nuclear atypia and cytoplasmic vacuolization to extensive stromal fibrosis. These treatment-related changes often obscure original tumor characteristics, making standard histological grading challenging and necessitating standardized evaluation systems like the Residual Cancer Burden (RCB) index to accurately quantify residual disease [11,23]. However, the histological grade continues to be a predictive factor during neoadjuvant therapy and should be included in the report. Nevertheless, the relative contribution of traditional histopathological parameters and IHC biomarkers to RCB classification, particularly in multivariable contexts, remains incompletely defined.

While the prognostic value of proliferation markers like Ki67 and histological grade is well-established, they are not sufficient to fully stratify risk in breast cancer treated with neoadjuvant chemotherapy. A significant proportion of patients still experience extensive residual disease (RCB-III), yet standard biomarkers often fail to distinguish these resistant tumors from those that will respond. Therefore, we aimed to explore the relationships among the Nottingham Score, Lymphovascular Invasion (LVI), tumor necrosis, IHC markers (Ki67, ER, PR, and HER2), and tumor regression, as measured by RCB, in a cohort of breast cancer patients treated with neoadjuvant chemotherapy. We sought to evaluate whether these morphological features serve as independent predictors of extensive residual disease (RCB-III), thereby providing prognostic information beyond established proliferation indices. These findings could enhance pathological risk stratification and improve the accuracy of clinical decision-making.

## 2. Materials and Methods

A retrospective cohort study was conducted at the Domina Sana Pathology Clinic in Bucharest. This study included a total of 165 patients with non-metastatic ductal breast cancer who underwent neoadjuvant chemotherapy post-core needle biopsy, followed by definitive breast surgery from January 2019 to December 2022. Patients who presented with distant metastases and those who underwent neoadjuvant hormonal therapy were excluded from the study. Clinical, pathological, and treatment-related data were collected from medical records and pathology reports. The study was approved by the clinic’s research committee in 2023 (573/08.02.2023). This study followed the principles of the Helsinki Declaration. The requirement of patient-informed consent was waived because of its retrospective nature.

All tissue specimens were processed according to standard institutional protocols, formalin-fixed and paraffin-embedded, and sectioned at 4 μm thickness for routine Hematoxylin and Eosin (H&E) staining.

Histopathological tumor characteristics that influenced treatment responses were analyzed on pre-treatment core needle biopsies. These include Nottingham Score, necrosis, the presence of lymphovascular invasion, tumor-infiltrating lymphocytes (TILs), and post-treatment regression status (RCB score).

The Nottingham Score evaluates glandular/tubular differentiation, nuclear pleomorphism, and mitotic count. Scores for each component were summed to generate a total score ranging from 3 to 9, corresponding to grade I (3–5), grade II (6–7), and grade III (8–9).

To ensure the integrity of the Nottingham Grading System analysis, this study included only non-metastatic ductal (NST) breast cancer cases, as this grading system may overestimate the grade for other histological subtypes like lobular carcinoma with limited tubule formation.

TILs were assessed on pre-treatment core needle biopsies according to the recommendations of the International TILs Working Group [24]. TILs were expressed as the percentage of stromal area occupied by mononuclear inflammatory cells within the invasive tumor. Areas of necrosis, DCIS, and biopsy-related artifacts were excluded. TILs were quantified as a continuous percentage and subsequently classified into three categories for analysis.

- Low TILs: <10% stromal infiltration.
- Intermediate TILs: 10–40% stromal infiltration.
- High (Abundant) TILs: >40% stromal infiltration.

The RCB (Residual Cancer Burden) Scoring System is a classification system utilized to evaluate tumor regression in breast cancer following neoadjuvant treatment, varying from RCB-0 (full regression) to RCB-III (minimal or no regression) [7,14].

RCB-0 (pathological complete response): no residual invasive carcinoma in the breast or axillary lymph nodes (RCB index = 0).

RCB-I (minimal residual disease): near-complete response with only small foci of residual invasive carcinoma (RCB index > 0 to 1.36).

RCB-II (moderate residual disease): partial response with moderate residual tumor cellularity in the breast and/or involved lymph nodes (RCB index > 1.36 to 3.28).

RCB-III (extensive residual disease): chemoresistant disease with substantial residual tumor cellularity and/or extensive nodal involvement (RCB index > 3.28), as calculated using the MD Anderson RCB calculator.

The RCB index was calculated for all patients using the standard MD Anderson Cancer Center web-based calculator. The RCB score was derived from the following five pathological parameters assessed on post-treatment surgical specimens:

1. Primary tumor bed area;
2. Overall cancer cellularity within the tumor bed (percentage);
3. Percentage of carcinoma in situ;
4. Number of positive lymph nodes;
5. Diameter of the largest lymph node metastasis.

Post-neoadjuvant pathological staging (ypStage) was also assessed according to the AJCC 8th Edition criteria [6].

Immunohistochemistry examination was conducted at the Domina Sana Pathology Clinic in Bucharest utilizing specific antibodies against ER, PR, KI67, and HER2/neu from Biocare Medical as listed in Table 1.

Scoring included only the invasive tumor, excluding the in situ area on the slides. The internal control was based on the normal glandular breast tissue. Hormone receptor status (ER and PR) was evaluated using the semi-quantitative Allred scoring system, which calculates a Total Score (TS) ranging from 0 to 8 by summing the Proportion Score (PS) and Intensity Score (IS). The score is calculated by adding the percentage of positive cells (0–0; 0–1%: 1; 1–10%: 2; 10–33%: 3; 33–66%: 4; 66–100%: 5) to the nuclear marking intensity (Absent—0; Weak—1; Moderate—2; Strong—3). Tumors with an Allred score of 2 or less were classified as negative, whereas those with a score of more than 2 were declared positive. This cutoff was selected to ensure concordance with the current ASCO/CAP guidelines, as any sample with ≥1% positive cells (equivalent to Allred PS ≥ 2) exhibits a minimum Total Score of 3 (PS 2 + IS 1). Consequently, tumors with a Total Score of 0–2 were classified as negative (corresponding to <1% staining), and tumors with a Total Score of 3–8 were classified as positive. To ensure absolute compliance with current standards, a secondary review of all original slides was performed; this confirmed that all of cases met the ASCO/CAP threshold, and no cases required reclassification. Scoring for HER2 expression was conducted using a 0–3 scale. Specimens showing a HER2 3+ staining score were classified as positive, indicating intense and full membrane staining in over 10% of cells. Specimens with HER2 staining scores of 0 and 1+ were classified as negative, indicating little to no membrane staining in less than 10% of tumor cells. Specimens with a score of 2+ (showing weak to moderate complete membrane staining in over 10% of tumor cells or strong complete membrane staining in less than 10% of tumor cells) were classified as equivocal. The specimens were evaluated using chromogenic in situ hybridization with the CISH Kit from Biocare Medical, (Biocare Medical, LLC, Pacheco, CA, USA) and only cases showing gene amplification were classified as positive. The HER2 gene was considered amplified if more than five signals per nucleus were identified, or a cluster of amplified signals per nucleus was present in over 50% of tumor cells. For the purpose of regression analyses, final HER2 status was coded as positive if IHC 3+ or CISH-amplified (*n* = 23, 13.94%), and negative otherwise (IHC 0, 1+, or 2+ without amplification). Ki67 expression was assessed as the percentage of positively stained tumor nuclei in hot spot areas, scoring at least 500 tumor cells across representative high-power fields (40×), and analyzed as a continuous variable in all regression models. No formal interobserver reproducibility metric (e.g., kappa statistic) was calculated; however, discordant cases were resolved by consensus review with a third pathologist (M.C.), as described above. For the purpose of intrinsic subtype classification only, Ki67 was dichotomized at the St. Gallen-recommended cutoff of 20%, but was analyzed continuously in regression.

For the purpose of this study, all original hematoxylin and eosin and immunohistochemical-stained slides from pre-treatment core biopsies and post-neoadjuvant surgical resection specimens were retrieved from the departmental archives. The review was conducted blinded to clinical outcomes and pathological response rates. Two pathologists (B.A.C and D.A.Ț.) individually performed these analyses, and the differences in results were settled by consulting a third pathologist (M.C.).

Breast cancer intrinsic subtypes were approximated using immunohistochemical surrogates according to the St. Gallen International Expert Consensus (2013/2015) [25]. Subtypes were defined as follows:

- Luminal A-like: ER+ and/or PR+, HER2−, Ki67 low (<20%).
- Luminal B-like (HER2-negative): ER+ and/or PR+, HER2−, and either Ki67 high (≥20%) or PR negative/low.
- Luminal B-like (HER2-positive): ER+ and/or PR+, HER2+ (any Ki67).
- HER2-enriched: ER−, PR−, HER2+.
- Triple Negative (TNBC): ER−, PR−, HER2−.

Descriptive statistics were used to summarize the patient and tumor characteristics. Categorical variables were expressed as absolute numbers and percentages, while continuous variables were reported as means and standard deviations. Univariate logistic regression was performed to identify variables associated with RCB III. Variables with a *p*-value < 0.05 in univariate analysis were pre-specified for inclusion in multivariable logistic regression models. Additionally, clinically relevant covariates (Age and HER2 status) were retained in the multivariable model regardless of univariate significance, in order to control for known confounders. Necrosis was also retained to assess its independent contribution beyond univariate screening. This approach ensured both statistical rigor and clinical interpretability. To address overfitting, we performed Firth’s penalized likelihood regression (Supplementary Table S1). Model stability was confirmed using bootstrap resampling (500 iterations), yielding a stable AUC. Multicollinearity among the independent variables was evaluated using Variance Inflation Factors (VIFs). No missing data were present for the primary variables analyzed.

The primary endpoint was dichotomized as RCB-III versus RCB-0/I/II based on the clinical rationale that extensive residual disease (RCB-III) represents the most treatment-resistant category and carries the worst long-term prognostic implications, as demonstrated by Yau et al. [22]. While RCB is inherently an ordinal outcome, binary logistic regression was selected to maximize clinical interpretability and statistical power for detecting predictors of the most clinically relevant endpoint. To ensure that this dichotomization did not distort the findings or discard critical ordinal information, a sensitivity analysis was performed using an alternative response threshold (Good Response [RCB-0/I] vs. Poor Response [RCB-II/III]). As a sensitivity analysis, ordinal logistic regression across all four RCB categories was also performed and yielded concordant results (Supplementary Table S2). Correlations between histopathological and immunohistochemical variables were assessed using Spearman’s correlation coefficients. Spearman’s rank correlation was selected as appropriate for the ordinal (Nottingham Score, TILs) and non-normally distributed continuous variables (Ki67) in this dataset. Binary variables (ER, PR, HER2 status, LVI, and necrosis) were coded as 0/1 for correlation analysis. Odds ratios (ORs) with corresponding 95% confidence intervals (CIs) were reported. A two-sided *p*-value < 0.05 was considered statistically significant. Statistical analyses were performed using SPSS version 26.0 (IBM Corp., Armonk, NY, USA) and GraphPad Prism version 10.6.1 (Graphpad Software Inc., San Diego, CA, USA).

## 3. Results

### 3.1. Baseline Characteristics and Morphological and Immunohistochemical Features Before Neoadjuvant Therapy

A total of 165 patients with stage I–III NST breast cancer treated with neoadjuvant were included in the analysis. The mean age at diagnosis was 63.3 years (SD = 13; range: 23–89 years).

**Figure 1:**
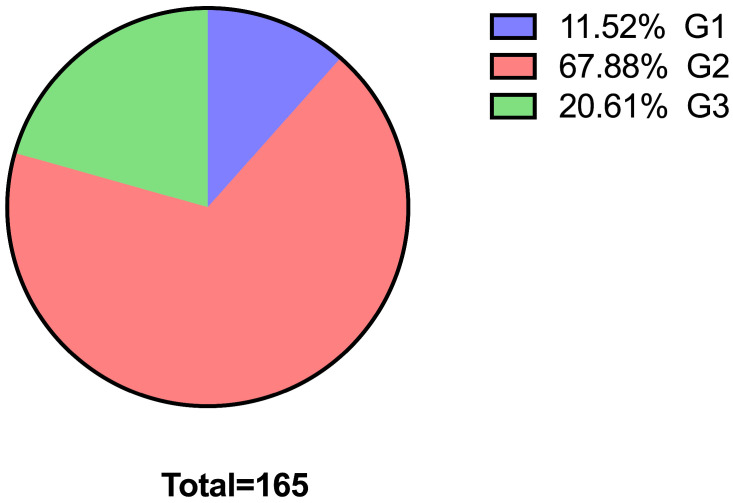
Tumor grade on core needle biopsy before NAC.

The core needle biopsies of the 165 cases revealed that most of the tumors (*n* = 112) were moderately differentiated based on the Nottingham score, followed by poorly differentiated tumors (*n* = 34), while only 19 tumors were well differentiated (Figure 1).

Additionally, we evaluated the presence of necrosis, lymphovascular invasion, TILs, and the expression of the immunohistochemical markers. The clinicopathological characteristics of the patients are presented in Table 2.

Following the CISH analysis, eight of the 28 equivocal HER2 cases presented gene amplification. As a consequence, the total number of HER2-positive cases was 23 (13.94%). Supplementary Table S3 details the IHC-to-CISH reclassification workflow.

Regarding the proliferation marker, Ki67, the median value was 30% (range: 5–90%). When stratified by the St. Gallen cutoff (≥20%), 76.64% of patients were classified as having high proliferative activity.

#### Correlation Analysis Between Histopathological and Immunohistochemical Variables

**Figure 2:**
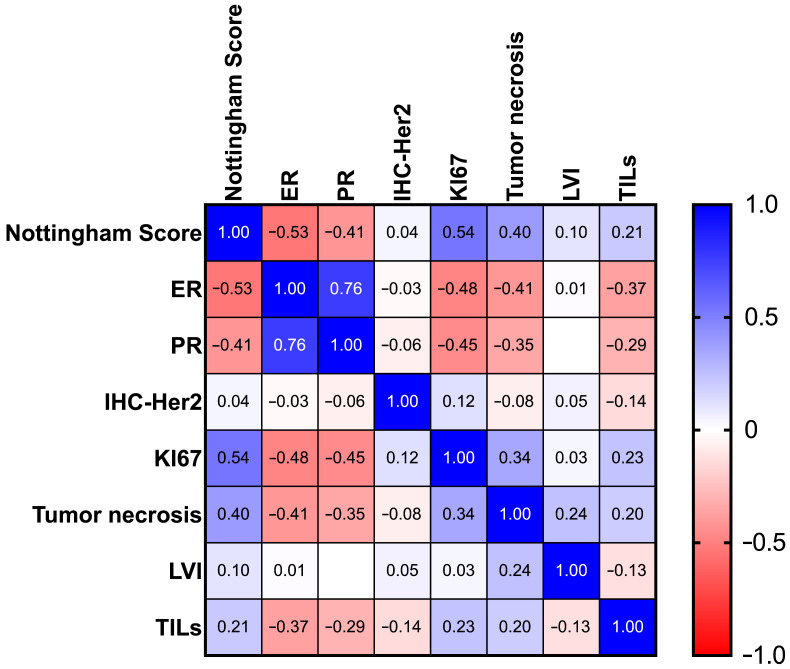
The Spearman Correlation Matrix coefficient values.

Spearman’s correlation analysis was performed to explore the relationships between Nottingham score, immunohistochemical markers, the presence of lymphovascular invasion, TILs and tumoral necrosis (Figure 2).

The *p*-values and the 95% CI of the Spearman Correlation Matrix are presented in Table 3.

Nottingham score demonstrated strong and significant positive correlations with both Ki67 expression and tumoral necrosis. Conversely, the Nottingham score was negatively and significantly correlated with ER and PR expression.

A strong positive correlation was also observed between ER and PR expression. Both ER and PR were significantly inversely correlated with Ki67 and with tumoral necrosis.

HER2 expression demonstrated weak and non-significant correlations with Nottingham score, hormone receptor status, Ki67, and tumoral necrosis, indicating relative independence from the other pathological variables assessed in this cohort.

Interestingly, the presence of lymphovascular invasion was only significantly, albeit weakly, correlated to tumor necrosis. Conversely, TILs were significantly positively correlated with the Nottingham score, Ki67 and tumoral necrosis and significantly negatively correlated with ER and PR expression.

### 3.2. Treatment Characteristics and Pathologic Response

**Figure 3:**
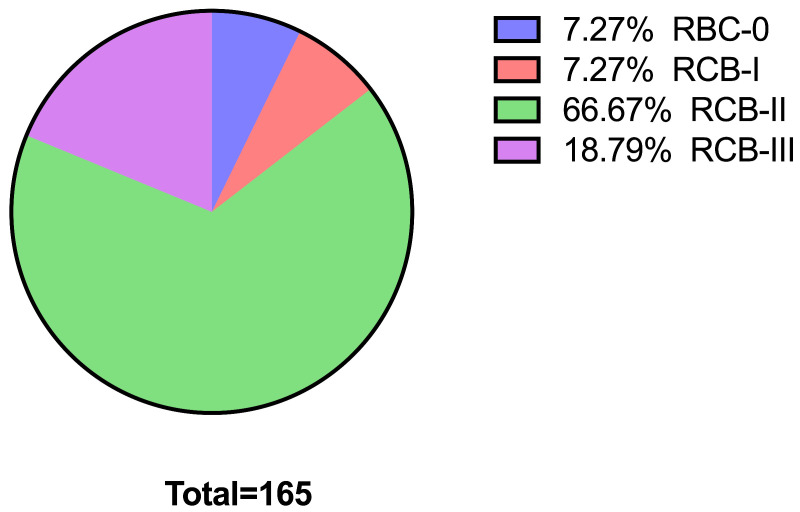
Proportion of RCB categories post-NAC.

The RCB post-NAC was classified as complete (RCB-0) in 7.27% (*n* = 12) of the patients. Similar numbers were noted for RCB-I. Patients with RCB-II (*n* = 110) represented the largest proportion (66.67%), while 18.79% of the patients (*n* = 31) displayed no regression (Figure 3).

**Figure 4:**
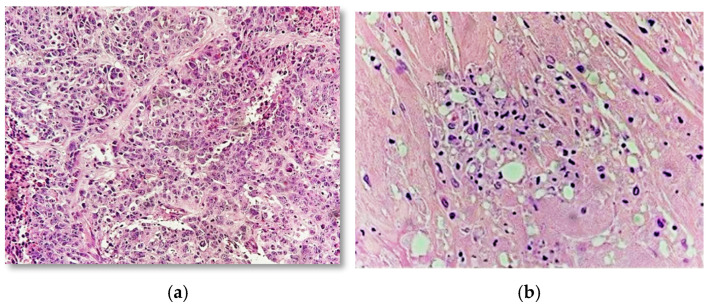
(**a**) High-grade invasive ductal carcinoma with no regression post-NAC (RCB-III) (H&E, 4×); (**b**) Complete regression (RCB-0) characterized by inflammatory cells, stromal fibrosis and cellular apoptosis with vacuolization (H&E, 10×).

Histopathological examination of post-treatment specimens revealed variable degrees of tumor regression. In cases with minimal or no response, residual tumor cells demonstrated preserved cellularity with limited morphological alteration. In contrast, responding tumors showed reduced cellularity, stromal fibrosis, increased collagen deposition, inflammatory infiltrates, and apoptotic changes. In cases of complete regression (RCB-0), the residual tumor was replaced by loose fibroelastotic tissue with scattered inflammatory cells and macrophages, consistent with treatment-related changes (Figure 4).

In this context, post-neoadjuvant pathological staging (ypStage) was also assessed according to the AJCC 8th Edition criteria (Table 4).

### 3.3. Univariate Analysis of Factors Associated with RCB III

Univariate logistic regression analysis was performed to identify clinicopathological and immunohistochemical factors associated with RCB III (Table 5).

As highlighted in Table 5, the age of the patients was not a significant predictor for RCB III. On the contrary, a higher Nottingham score was significantly associated with an increased likelihood of RCB III.

Among the immunohistochemical markers, an elevated Ki67 expression was significantly associated with RCB III. Estrogen receptor (ER) expression also demonstrated a statistically significant association with RCB III.

In contrast, tumoral necrosis, progesterone receptor (PR) status, and HER2 expression were not significantly associated with RCB III in univariate analysis.

Given the small number of cases in the high TIL category (*n* = 3, 1.82%), the TIL-associated regression estimates should be interpreted as descriptive rather than inferential, and this variable was not included in the multivariable model.

As an exploratory analysis, we approximated intrinsic molecular subtypes using immunohistochemical surrogates and analyzed their association with treatment response. Given the small sample sizes in several subtype categories, these results should be interpreted with caution and considered hypothesis-generating rather than confirmatory (Table 6).

Interestingly, the Triple-Negative subtype exhibited the highest proportion of extensive residual disease (RCB-III), with 38.9% of patients falling into this high-risk category. In contrast, Luminal A-like tumors rarely presented with extensive residual burden (2.8% RCB-III). The Luminal B-like (HER2−) subgroup, which constituted the majority of the cohort (53.9%), showed an intermediate response pattern, with 21.3% of patients exhibiting extensive residual disease. In the univariate analysis, intrinsic subtypes were significantly associated with the risk of extensive residual disease (RCB-III). Using the Luminal A-like subtype as the reference (baseline risk), patients with TNBC had a significantly higher likelihood of harboring extensive residual disease (OR 22.27; 95% CI 3.42–145.23; *p* = 0.001) in cases where pathological complete response was not achieved. Similarly, the Luminal B-like (HER2−) subtype was associated with a 9.5-fold increased risk of extensive residual burden compared to Luminal A tumors (*p* = 0.013). While HER2-enriched and Luminal B (HER2+) subtypes also showed elevated Odds Ratios, these did not reach statistical significance, likely due to the smaller sample sizes in these subgroups. Reflecting the limited precision of these small subgroups, the confidence intervals were notably wide, and these findings are intended to be interpreted as preliminary trends.

To evaluate the predictive performance of Ki67 within different biological contexts, we calculated the Area Under the Receiver Operating Characteristic curve (AUC) for each molecular subtype (Supplementary Table S4). Ki67 demonstrated strong discriminative ability for RCB-III in the Luminal cohort (AUC 0.76; 95% CI 0.68–0.84). While the smaller sample sizes in the HER2-positive and TNBC subgroups resulted in wider confidence intervals, the AUC values (0.68 and 0.71, respectively) remained directionally consistent with the primary findings, suggesting that Ki67 maintains its biological relevance as a marker of treatment resistance across various subtypes.

### 3.4. Multivariable Analysis of Factors Associated with RCB III

Subsequently, a multivariable logistic regression was performed to identify independent predictors of RCB-III (Table 7). Progesterone Receptor (PR) status was excluded from the multivariable logistic regression model due to its well-established collinearity with ER status.

In the multivariable analysis, Ki67 and lymphovascular invasion remained the only independent predictors of RCB III, while the Nottingham score and ER expression did not retain statistical significance.

Given the number of covariates relative to the event rate (*n* = 31 RCB-III cases), a sensitivity analysis using Firth’s penalized likelihood regression was performed to assess model stability. The results (detailed in Supplementary Table S1) confirmed that Ki67 (OR 1.03, *p* = 0.042) and lymphovascular invasion (OR 6.33, *p* = 0.022) remained the only stable independent predictors of extensive residual disease. Furthermore, all included predictors demonstrated a VIF < 2.0 (range: 1.05–1.55), indicating that multicollinearity did not significantly impact the stability or interpretation of the multivariable model.

Lastly, to confirm that the dichotomization of the primary endpoint (RCB-III vs. non-RCB-III) did not result in a significant loss of clinical information, a sensitivity analysis was performed using ordinal logistic regression across the full four-tier RCB spectrum (0, I, II, and III). Consistent with the primary binary model, Ki67 (OR = 1.04, 95% CI: 1.02–1.06; *p* < 0.001) and LVI (OR = 4.85, 95% CI: 2.15–10.92; *p* < 0.001) remained the only independent predictors of worsening residual disease status (Supplementary Table S2). These results validate the robustness of our primary modeling strategy and confirm that Ki67 and LVI are consistent drivers of chemoresistance across all levels of tumor regression.

## 4. Discussion

Residual cancer burden (RCB) has emerged as one of the most robust post-neoadjuvant prognostic tools in breast cancer, being superior to traditional pathologic complete response in predicting long-term outcomes across molecular subtypes [20,22].

This study evaluated associations among traditional histopathological parameters, immunohistochemical biomarkers, and tumor regression after neoadjuvant chemotherapy in a cohort of patients with stage I–III breast cancer. Our findings show that although the Nottingham score and ER expression are associated with poor tumor regression in univariate analyses, Ki67 is the only independent predictor of extensive residual disease (RCB III) after multivariable adjustment.

From a methodological perspective, the observed correlations among the Nottingham score, hormone receptor status, Ki67, and necrosis underscore the importance of considering multicollinearity when constructing predictive models. In particular, the strong correlations between ER/PR and Ki67, and between ER/PR and Nottingham score, suggest that some of these markers capture overlapping aspects of tumor biology. As a consequence, PR was excluded from the multivariable analysis. Nottingham score was significantly associated with RCB III in univariate analysis but lost significance after adjustment for Ki67. This finding suggests that the prognostic impact of histologic grade in the neoadjuvant setting may be largely mediated through its correlation with proliferative activity. These interdependencies indicate that multiple pathological variables capture overlapping aspects of tumor biology, and they help explain why morphology-based metrics may not retain independent predictive value in multivariable models. Spearman’s correlation analysis also revealed significant associations between stromal TILs and markers of tumor aggression. We observed a significant positive correlation between TIL density and Nottingham histological grade, Ki67 proliferation index, and the presence of tumor necrosis. Conversely, TILs were negatively correlated with hormone receptor (ER and PR) expression. These findings align with the established paradigm that higher immunogenicity is associated with aggressive biological phenotypes [26,27]. Highly proliferative and high-grade tumors are known to harbor greater genomic instability and mutational burden, leading to increased neoantigen presentation and subsequent immune recruitment [28]. Furthermore, the inverse relationship with ER/PR expression confirms that hormone-receptor-positive breast cancers are generally less immunogenic (‘cold tumors’) compared to their hormone-receptor-negative counterparts, highlighting the biological distinctiveness of the immune microenvironment across subtypes [24,29]. In this context, TIL density itself did not serve as a predictor of pathological response in our univariate analysis. This finding is consistent with the distinct immunobiology of hormone-receptor-positive breast cancer. Unlike Triple-Negative or HER2-positive subtypes, where high TIL levels are strongly predictive of pathological complete response (pCR) and improved survival, the prognostic value of TILs in Luminal breast cancer remains controversial and less consistent. In hormone-receptor-positive tumors, the immune response is often dampened by lower mutational burdens and the immunosuppressive effects of estrogen signaling. Consequently, lower absolute levels of TILs in Luminal tumors may be insufficient to generate a robust anti-tumor immune response during chemotherapy, consistent with the findings that higher TIL concentrations are required to significantly improve pathologic complete response rates [26]. Furthermore, from a statistical perspective, the significant correlation we observed between TILs and other prognostic variables (Nottingham Grade and Ki67) indicates a high degree of multicollinearity. Since TILs failed to demonstrate independent predictive value in the univariate setting and tracked closely with established proliferative markers, they were excluded from the final multivariable model. This exclusion was necessary to preserve statistical power and prevent model overfitting, ensuring that the identified predictors (such as LVI) represent truly independent drivers of treatment resistance in this cohort.

We also identified a statistically significant, albeit weak, positive correlation between Lymphovascular Invasion (LVI) and tumor necrosis. This association reinforces the link between rapid tumor proliferation and invasive potential. Biologically, tumor necrosis is a hallmark of hypoxia, occurring when rapid neoplastic growth outstrips the vascular supply [30]. The hypoxic microenvironment is a known driver of angiogenesis and lymphangiogenesis via the upregulation of factors such as VEGF-C, which in turn facilitates the entry of tumor cells into the vascular and lymphatic channels [31,32]. The weak nature of the correlation suggests that while necrosis and LVI share common drivers of aggressiveness, they represent distinct biological events—necrosis reflecting metabolic stress and LVI reflecting metastatic motility—that do not always occur simultaneously in luminal breast cancer. Most notably, our multivariable analysis identified Lymphovascular Invasion (LVI) as a robust, independent predictor of extensive residual disease (RCB-III). This finding highlights that LVI captures a dimension of tumor aggressiveness—specifically, metastatic proficiency—that is not fully represented by cellular proliferation markers alone. The presence of LVI indicates that tumor cells have successfully detached from the primary mass, degraded the extracellular matrix, and gained entry into the systemic circulation, marking the critical first step in the metastatic cascade [33]. Previous studies have consistently demonstrated that LVI is associated with higher rates of axillary lymph node involvement and poorer disease-free survival in breast cancer patients receiving neoadjuvant chemotherapy [34,35]. However, it is important to acknowledge that LVI assessment on core needle biopsies is inherently susceptible to sampling bias and underdetection, particularly in small pre-treatment specimens. The absence of LVI on biopsy does not equate to its biological absence within the tumor, and this limitation should temper the clinical weight assigned to this variable as a standalone decision marker. With this caveat, our data suggest that the presence of LVI in the pre-treatment biopsy may serve as a morphological surrogate for a ‘chemo-resistant’ phenotype, possibly due to the overlap between invasive motility pathways and drug-resistance mechanisms [36]. While these findings are noteworthy, LVI on core biopsy should be interpreted as one component within a broader risk stratification framework rather than as a standalone clinical decision marker.

The association between high Ki67 expression and poor tumor regression underscores the central role of tumor proliferation in shaping response to neoadjuvant chemotherapy. Ki67 reflects the fraction of actively cycling tumor cells and is therefore mechanistically linked to the efficacy of cytotoxic agents that primarily target dividing cells, being a significant predictor for better RCB [19,37,38,39]. Although high proliferative tumors are often considered more chemosensitive, our results suggest that elevated Ki67 may also identify biologically aggressive tumors that retain substantial residual disease despite systemic therapy. In this context, Lee SY et al. also showed that elevated baseline Ki67 is also associated with aggressive tumor biology and higher residual disease burden [40]. This apparent paradox highlights the complexity of treatment response and supports the interpretation of Ki67 as a marker of intrinsic tumor aggressiveness rather than a straightforward predictor of chemosensitivity.

The retention of Ki67 as an independent predictor of poor tumor regression suggests that proliferation-based markers may provide a more direct reflection of tumor behavior than composite grading systems in the neoadjuvant setting. Previous studies have demonstrated strong correlations between histologic grade and Ki67 expression, indicating that these variables capture overlapping dimensions of tumor biology [3,16]. In the neoadjuvant setting, where treatment response is driven largely by cellular proliferation and chemosensitivity, proliferation-based biomarkers may therefore supersede morphology-based grading systems in predictive models. These results align with prior reports suggesting that while histologic grade remains prognostically relevant in untreated disease, its independent predictive value may be attenuated when more direct measures of tumor biology are included in multivariable analyses [15,22]. Nevertheless, the loss of significance for the histological grade in the multivariable model could be partly due to biological collinearity with Ki67, as high-grade tumors typically exhibit elevated proliferation rates.

Hormone receptor expression showed expected biological patterns, with strong correlation between ER and PR positivity and inverse relationships with Ki67 and tumoral necrosis, reflecting the well-established biological distinction between hormone receptor-positive, luminal-type tumors and highly proliferative, poorly differentiated tumors [3,4]. While the lack of expression of ER was a negative prognostic factor in the univariate analysis, neither ER nor PR status independently predicted tumor regression in the multivariable model. This finding is consistent with previous studies demonstrating that hormone receptor status alone is an imperfect predictor of response to neoadjuvant chemotherapy, particularly when proliferation markers are accounted for [16,17]. Our data suggest that proliferative activity, rather than hormone receptor expression per se, is the dominant determinant of poor tumor regression in this cohort.

HER2-positive breast cancer is generally associated with high chemosensitivity, particularly in the era of HER2-targeted therapies. Large pooled analyses, such as that by Yau c et al. have demonstrated favorable outcomes in HER2-positive tumors achieving low RCB scores following NAC [22]. However, HER2 expression did not show a significant association with tumor regression or RCB class in our study. This finding contrasts with reports of high chemosensitivity in HER2-positive breast cancer, particularly in the context of HER2-targeted therapy. The lack of association observed here may reflect sample size limitations, treatment heterogeneity, or incomplete stratification based on anti-HER2 agents. Solomon JP et al. emphasized that accurate HER2 assessment and treatment context are essential when interpreting response data [41]. Consequently, the lack of association observed here should be interpreted cautiously and does not invalidate the established predictive value of HER2 in appropriately treated populations.

Our analysis of intrinsic subtypes highlights the polarized nature of response in aggressive phenotypes. Consistent with large meta-analyses [39,40], who demonstrated that residual burden in aggressive subtypes carries a distinct and more severe prognostic weight compared to luminal disease, we observed that Triple-Negative Breast Cancer exhibited the highest rates of extensive residual disease. Specifically, TNBC patients had the highest risk of extensive residual disease (RCB-III, 38.9%) compared to Luminal A patients (OR 22.27, *p* = 0.001) among those who failed to achieve a complete response. While large meta-analyses [42] highlight that TNBC achieves the highest rates of pathological complete response (pCR), our data reflect the ‘all-or-nothing’ nature of chemotherapeutic response in this subtype. Conversely, Luminal A-like tumors had the lowest RCB-III rate (2.8%). Despite their relative resistance to chemotherapy (lower pCR rates), they rarely presented with extensive residual burden (RCB-III), likely due to their lower proliferation rates (low Ki67), which may prevent the rapid accumulation of extensive resistant tumor mass during treatment. While our primary analysis established Ki67 as a robust predictor for the entire cohort, the exploratory subtype-stratified analysis (Table 6 and Supplemental Table S4) suggests that this relationship persists across different biological backgrounds. However, we acknowledge that the small sample sizes in the HER2-positive (*n* = 23) and TNBC (*n* = 18) subgroups limit the statistical power of these specific observations, as reflected by the wider confidence intervals. These findings should therefore be interpreted as hypothesis-generating rather than confirmatory. They suggest a biological trend where high proliferation remains a marker of chemotherapy sensitivity even in subtypes typically driven by targeted pathways, but these results require validation in larger, multi-center cohorts.

From a hypothesis-generating perspective, the identification of Ki67 as an independent predictor of poor tumor regression may warrant further investigation regarding its potential integration into response prediction frameworks. However, given the retrospective, single-center nature of this study and the limited sample size, these findings should not be interpreted as directly supporting clinical implementation of response-adapted intensification or de-escalation strategies. As emphasized by ASCO guidelines, any such strategies require rigorous prospective validation before routine clinical adoption [15].

In spite of the significant findings presented in this study, several limitations should also be acknowledged. First, the retrospective design with a restricted sample size introduces the potential for selection bias and limits causal inference. For instance, we acknowledge that the reprospective HER2 assessment was based on the IHC042 clone and CISH criteria that differ slightly from the ASCO CAP 2023 recommendations. While this reflects our institutional diagnostic standards during the study period (2019–2022), we recognize that variations in clone sensitivity or interpretation thresholds could influence the classification of equivocal cases. However, this is counterbalanced by the centralized pathological review of all specimens, which ensures high data consistency and allows for the precise evaluation of morphological markers like LVI and TILs, which are not routinely captured in large cancer registries. Second, this paper does not address the prognostic value of other histological subtypes of breast cancer apart from NST carcinomas. However, other subtypes were excluded in order to maintain the diagnostic consistency of the Nottingham Grading System, as this scoring system is not the standard of care for lobular or other special-type carcinomas. Third, with 31 RCB-III events (18.8% of the cohort), the multivariable model including six or more covariates yields fewer than six events per variable, which falls below the recommended threshold of ten events per parameter and increases the risk of overfitting. Consequently, the retained significance of Ki67 and LVI should be interpreted with caution, particularly given the wide confidence intervals observed for LVI (OR 0.023–0.626). However, Bootstrap analysis confirmed predictor stability (500 resamples; Ki67 AUC stability 0.87 ± 0.04) but future validation in larger cohorts is essential to confirm predictor stability. Fourth, treatment heterogeneity represents an important limitation. The 2019–2022 cohort spans varying neoadjuvant chemotherapy regimens without stratification by chemotherapy type, anti-HER2 therapy, or treatment line. This is particularly relevant for HER2-positive cases (*n* = 23, 13.94%) and TNBC cases (*n* = 18, 10.9%), where response varies substantially by regimen. However, the majority of the cohort (86%) comprised Luminal subtypes, which are generally less regimen-sensitive. A detailed treatment table including regimen type (anthracycline/taxane-based, platinum-containing), anti-HER2 therapy use, and number of cycles was not available for the present analysis and represents a critical gap. Future studies should standardize NAC protocols or stratify analyses by specific therapeutic regimens to better control for this confounding factor. Fifth, key pretreatment clinical variables, including radiological tumor diameter, clinical T and N stage (cT/cN), menopausal status, and grade distribution stratified by molecular subtype, were not systematically recorded in the pathology database and could not be included in this analysis. This limits the interpretability of residual disease patterns across subtypes and represents an important area for improvement in future prospective studies. Sixth, Ki67 assessment is subject to interobserver variability and methodological differences, which may affect reproducibility across institutions. Furthermore, LVI was assessed using standard H&E staining without the use of confirmatory immunohistochemical markers. While this reflects routine diagnostic practice and was mitigated by a triple-observer consensus (including a third senior pathologist for discrepancies), we acknowledge that the lack of IHC may influence the detection of subtle vascular involvement or lead to the misinterpretation of retraction artifacts. Additionally, given the loss of significance for other parameters in multivariable analysis, caution is warranted before implementing clinical decision-making based solely on these markers. While individual markers showed promise in univariate analysis, their overlapping biological information limits their independent predictive utility when assessed simultaneously. These results underscore the need for integrated, multivariable predictive models that account for marker interdependence. Future research should focus on developing and validating composite scoring systems or molecular classifiers that capture the full spectrum of tumor biology relevant to treatment response. Prospective validation in larger cohorts with standardized treatment protocols and long-term outcome data will be essential to translate these pathological findings into clinically actionable tools that can guide personalized treatment decisions and improve patient outcomes in the neoadjuvant setting. Finally, long-term clinical outcomes such as disease-free and overall survival were not available, precluding direct correlation between RCB classification and patient prognosis.

## 5. Conclusions

In conclusion, this study identifies Ki67 and lymphovascular invasion as independent predictors of extensive residual disease following neoadjuvant chemotherapy in breast cancer. While Nottingham score and ER expression were associated with tumor regression in univariate analysis, their predictive value diminished after adjustment for proliferative activity, suggesting that proliferation-based markers may capture overlapping biological information. These findings are hypothesis-generating and require confirmation in larger, multicenter cohorts with standardized treatment protocols.

These findings support the integration of proliferation markers into multivariable predictive models for response assessment in the neoadjuvant setting. Rather than relying on single pathological parameters, future approaches should account for the interdependence of morphological and molecular features. The limited sample size, single-center design, absence of detailed treatment stratification, and lack of long-term survival data preclude definitive clinical recommendations at this stage. Prospective validation in larger, well-characterized cohorts with standardized treatment protocols and long-term outcome data will be essential before translating these findings into clinical practice.

## Figures and Tables

**Table 1:** Immunohistochemical markers’ specifications.

Antibody	Clone	Source
Estrogen receptor	SP1	Rabbit monoclonal
Progesterone receptor	SP2	Rabbit monoclonal
HER2/neu	IHC042	Mouse monoclonal
Ki67	SP6	Rabbit monoclonal

**Table 2:** The clinico-pathological characteristics of the patients.

Variable	Pattern	Total
Age	Mean: 63.3 years (SD = 13)	43.64% (*n* = 72) ≤63
	Range: 23–89	56.36% (*n* = 93) >63
Tumor necrosis	Present	20.61% (*n* = 34)
	Absent	79.39% (*n* = 131)
Lymphovascular invasion	Present	23.03% (*n* = 38)
	Absent	76.97% (*n* = 127)
Tumor-infiltrating lymphocytes	Low (<10%)	82.42% (*n* = 136)
	Intermediate (10–40%)	15.76% (*n* = 26)
	High (>40%)	1.82% (*n* = 3)
ER	Positive	84.85% (*n* = 140)
	Negative	15.15% (*n* = 25)
PR	Positive	76.36% (*n* = 126)
	Negative	23.64% (*n* = 39)
HER2	Positive	9.09% (*n* = 15)
	Negative	73.94% (*n* = 122)
	Equivocal	16.97% (*n* = 28)
Ki67	Low	23.64% (*n* = 39)
	High	76.36% (*n* = 126)

**Table 3:** The *p*-values and the 95% CI of the Spearman Correlations.

	Nottingham Score		ER		PR		HER2		Ki67		Tumor Necrosis		LVI		TILs	
	*p*-Value	95% CI	*p*-Value	95% CI	*p*-Value	95% CI	*p*-Value	95% CI	*p*-Value	95% CI	*p*-Value	95% CI	*p*-Value	95% CI	*p*-Value	95% CI
**Nottingham score**			<0.0001	−0.64–−0.41	<0.0001	−0.54–−0.27	0.605	−0.12–0.2	<0.0001	0.41–0.64	<0.0001	0.25–0.52	0.186	−0.05–0.26	0.007	0.05–0.35
**ER**	<0.0001	−0.64–−0.41			<0.0001	0.68–0.82	0.749	−0.18–0.13	<0.0001	−0.59–−0.35	<0.0001	−0.53–−0.27	0.901	−0.15–0.17	<0.0001	−0.5–−0.23
**PR**	<0.0001	−0.54–−0.27	<0.0001	0.68–0.82			0.411	−0.22–0.09	<0.0001	−0.57–−0.32	<0.0001	−0.48–−0.21	0.994	−0.16–0.16	<0.0001	−0.43–−0.14
**HER2**	0.6404	−0.12–0.2	0.7485	−0.18–0.13	0.4111	−0.22–0.09			0.119	−0.04–0.27	0.3367	−0.23–0.08	0.492	−0.1–0.21	0.072	−0.29–0.02
**Ki67**	<0.0001	0.42–0.64	<0.0001	−0.59–−0.35	<0.0001	−0.57–−0.32	0.119	−0.04–0.27			<0.0001	0.2–0.48	0.669	−0.12–0.19	0.003	0.08–0.38
**Tumor** **Necrosis**	<0.0001	0.25–0.52	<0.0001	−0.53–−0.27	<0.0001	−0.48–−0.21	0.337	−0.23–0.08	<0.0001	0.2–0.48			0.002	0.09–0.39	0.011	0.04–0.34
**LVI**	0.1864	−0.05–0.26	0.9012	−0.15–0.167	0.9937	−0.16–0.16	0.492	−0.1–0.21	0.6692	−0.12–0.19	0.0016	0.09–0.39			0.109	−0.28–0.03
**TILs**	0.0073	0.05–0.35	<0.0001	−0.5–−0.23	0.0001	−0.43–−0.14	0.072	−0.29–0.02	0.0025	0.08–0.38	0.0114	0.04–0.34	0.109	−0.28–0.03		

**Table 4:** Post-neoadjuvant pathological staging.

Post-Neoadjuvant Pathological Stage (ypStage)	Total
Stage 0	7.3% (*n* = 12)
Stage I	44.2% (*n* = 73)
Stage II	29.7% (*n* = 49)
Stage III	18.8% (*n* = 31)

**Table 5:** Univariate Logistic Regression of Factors Associated with RCB III.

Variable		OR	95% CI	*p*-Value
Age		1.017	0.9864–1.051	0.2845
Nottingham score		1.807	1.246–2.678	0.0017
ER (negative)		3.017	1.152–7.611	0.0255
PR (positive)		0.4803	0.2082–1.145	0.0961
HER2 (positive)		0.8967	0.2451–2.625	0.8522
Ki67		1.034	1.015–1.054	0.0003
Necrosis		1.784	0.7067–4.256	0.2127
Lymphovascular invasion (absent)		0.1877	0.02942–0.668	0.0069
TILs	Low	Reference		
	Intermediate	1.81	0.6458–4.662	0.247
	High	2.457	0.1115–26.69	0.4958

**Table 6:** Exploratory Association between Intrinsic Molecular Subtypes and Residual Cancer Burden.

Molecular Subtype	Total	Good/Intermediate Response (RCB 0–II)	Extensive Residual Disease (RCB III)	OR	95% CI	*p*-Value
Luminal A-like	21.8% (*n* = 36)	97.2% (*n* = 35)	2.8% (*n* = 1)	Reference	-	-
Luminal B-like (HER2−)	53.9% (*n* = 89)	78.7% (*n* = 70)	21.3% (*n* = 19)	9.5	1.72–52.47	0.013
Luminal B-like (HER2+)	10.9% (*n* = 18)	83.3% (*n* = 15)	16.7% (*n* = 3)	7.00	0.94–51.91	0.103
HER2-enriched	2.4% (*n* = 4)	75.0% (*n* = 3)	25.0% (*n* = 1)	11.67	0.94–144.36	0.192
TNBC	10.9% (*n* = 18)	61.1% (*n* = 11)	38.9% (*n* = 7)	22.27	3.43–145.23	0.001

**Table 7:** Multivariable Logistic Regression of Factors Associated with RCB III.

Variable	OR	95% CI	*p*-Value
Age	1.016	0.9827–1.052	0.3590
Nottingham score	1.318	0.7335–2.370	0.3531
Lymphovascular invasion (absent)	0.1579	0.023–0.626	0.0062
ER (negative)	1.518	0.3833–5.986	0.5483
HER2 (positive)	0.6347	0.1535–2.108	0.4755
Ki67	1.028	1.002–1.056	0.0363
Necrosis (present)	0.4465	0.1194–1.45	0.1861

## Data Availability

Data is contained within the article or Supplementary Materials.

## Supplementary Materials

The following supporting information can be downloaded at: https://www.mdpi.com/article/10.3390/diagnostics16081213/s1, Table S1: Multivariable Model Diagnostics: Firth’s Penalized Regression and Collinearity Assessment (VIF); Table S2: Sensitivity Analysis of Predictors across RCB Classes; Table S3: HER2 Status Reclassification: Correlation between Immunohistochemistry (IHC) and Chromogenic In Situ Hybridization (CISH); Table S4. Predictive Performance of Ki67 across Molecular Subtypes: Area Under the Curve (AUC) and 95% Confidence Intervals.

## Abbreviations

The following abbreviations are used in this manuscript:

AJCC	American Joint Committee on Cancer
ASCO/CAP	American Society of Clinical Oncology/College of American Pathologists
CI	Confidence Interval
CISH	Chromogenic In Situ Hybridization
ER	Estrogen Receptor
H&E	Hematoxylin and Eosin
HER2	Human Epidermal Growth Factor Receptor 2
IHC	Immunohistochemistry
LVI	Lymphovascular Invasion
NAC	Neoadjuvant chemotherapy
OR	Odds Ratio
pCR	Pathological Complete Response
RCB	Residual Cancer Burden
SD	Standard Deviation
TILs	Tumor-Infiltrating Lymphocytes
TNBC	Triple-Negative Breast Cancer
ypStage	Post-neoadjuvant Pathological Stage
