# Supplementary material for: Ki67 and Lymphovascular Invasion as Histopathological Predictors of Residual Cancer Burden After Neoadjuvant Chemotherapy in Breast Cancer: A Retrospective Study
Source: Diagnostics (Basel). 2026 Apr 18;16(8):1213. doi: 10.3390/diagnostics16081213 (PMC13114671; doi:10.3390/diagnostics16081213)
Supplement: Supplementary file 1 [file diagnostics-16-01213-s001.zip › diagnostics-4201638-supplementary.pdf]

**Supplementary Table S1.** Multivariable Model Diagnostics: Firth's Penalized Regression and Collinearity Assessment (VIF).

| Variable | Coefficient | Odds Ratio | p-value | VIF |
|---|---|---|---|---|
| Age | 0.0157 | 1.02 | 0.366 | 1.05 |
| Nottingham score | 0.276 | 1.32 | 0.352 | 1.48 |
| ER positive | -0.417 | 0.66 | 0.548 | 1.55 |
| HER2 positive | -0.455 | 0.63 | 0.488 | 1.1 |
| KI67 | 0.027 | 1.03 | 0.042 | 1.35 |
| LV invasion present | 1846 | 6.33 | 0.022 | 1.08 |
| Tumoral necrosis present | -0.806 | 0.45 | 0.202 | 1.15 |

**Supplementary Table S2.** Sensitivity Analysis of Predictors across RCB Classes.

| Variable | Odds Ratio | 95% Confidence Interval | p-value |
|---|---|---|---|
| **Ki67 (%)** | 1.04 | 1.02–1.06 | < 0.001 |
| **LVI (Presence)** | 4.85 | 2.15–10.92 | < 0.001 |
| **Age** | 0.99 | 0.96–1.02 | 0.612 |
| **ER Status** | 1.25 | 0.55–2.84 | 0.585 |
| **HER2 Status** | 0.78 | 0.32–1.91 | 0.589 |
| **Necrosis** | 1.12 | 0.48–2.61 | 0.784 |
| **Nottingham Grade** | 1.41 | 0.72–2.75 | 0.318 |
| Odds Ratios represent the likelihood of moving into a higher RCB category (e.g., from RCB-I to RCB-II) for every unit increase in the predictor. The consistency between this ordinal model and our binary RCB-III model confirms that the selection of RCB-III as the primary endpoint did not result in a significant loss of predictive information. | | | |

**Supplementary Table S3.** HER2 Status Reclassification: Correlation between Immunohistochemistry (IHC) and Chromogenic In Situ Hybridization (CISH).

| Initial IHC Category | Number of cases | CISH Result | Final HER2 Status (Used in Regression) |
|---|---|---|---|
| IHC 3+ (Positive) | 15 | Not required | 15 Positive |
| IHC 2+ (Equivocal) | 28 | 8 Amplified / 20 Not Amplified | 8 Positive / 20 Negative |
| IHC 0 / 1+ (Negative) | 122 | Not required | 122 Negative |
| Total Cohort | 165 | | 23 Positive (13.94%) |

**Supplementary Table S4.** Predictive Performance of Ki67 across Molecular Subtypes: Area Under the Curve (AUC) and 95% Confidence Intervals.

| Molecular Subtype | Total (n=165) | RCB-III Cases (n=31) | Median Ki67 (%) | AUC for Ki67 (95% CI) |
|---|---|---|---|---|
| Luminal (ER+/HER2-) | 142 (86.1%) | 24 | 25% | 0.76 (0.68–0.84) |
| HER2-Positive | 23 (13.9%) | 4 | 45% | 0.68 (0.45–0.91)* |
| TNBC | 18 (10.9%) | 3 | 70% | 0.71 (0.48–0.94)* |

*Note: Due to the small sample size in HER2+ and TNBC subgroups, p-values for these specific AUCs did not reach significance; however, the overall trend supports Ki67 as a biological driver of response.
